# Clinical Implications of Obstructed Hemivagina and Ipsilateral Renal Anomaly (OHVIRA) Syndrome in the Prepubertal Age Group

**DOI:** 10.1371/journal.pone.0166776

**Published:** 2016-11-18

**Authors:** Jang Hee Han, Yong Seung Lee, Young Jae Im, Sang Woon Kim, Mi-Jung Lee, Sang Won Han

**Affiliations:** 1 Department of Urology and Urological Science Institute, Yonsei University College of Medicine, Seoul, Korea; 2 Department of Urology, College of Medicine, Seoul National University, Seoul, Korea; 3 Department of Radiology, Yonsei University College of Medicine, Seoul, Korea; University of Kansas Medical Center, UNITED STATES

## Abstract

**Purpose:**

Obstructed hemivagina and ipsilateral renal anomaly (OHVIRA) syndrome is a rare syndrome characterized by Müllerian duct and renal anomalies. It is usually regarded as a disease of adolescence; however, due to a number of possible problems, the management of patients before puberty should not be overlooked. We assessed the clinical course of prepubertal patients to propose appropriate management.

**Materials and Methods:**

We retrospectively assessed 43 prepubertal OHVIRA syndrome patients who were diagnosed and followed up at our institution from July 2004 to June 2015. We reviewed medical records, focusing on presentation, radiologic findings, surgical management, and the overall clinical course.

**Results:**

Median age at diagnosis was 1.3 months and median follow-up period was 25.5 months. The most common accompanying ipsilateral urologic anomalies were ectopic ureter and ureterocele, while the most common contralateral anomaly was vesicoureteral reflux. During the follow-up period, six patients (14.0%) required surgery at a median age of 31.2 months due to recurrent urinary tract infection, uncontrolled vaginal distention compressing adjacent organs, urinary incontinence, or intractable abdominal pain.

**Conclusions:**

While OHVIRA syndrome is known as a postpubertal disease, about 13% of prepubertal patients in our study required surgery. When ectopic ureter insertion into the vagina is present, further treatment may be needed to address the complications caused by continuous urine production. Patients should be monitored for complications arising from either obstructed hemivagina or renal anomalies with regular follow-up, especially before the age of five years.

## Introduction

Obstructed hemivagina and ipsilateral renal anomaly (OHVIRA) syndrome, also known as Herlyn-Werner-Wunderlich syndrome, was first reported by Purslow in 1922 [1]. The pathogenesis of OHVIRA syndrome is related to anomalous development of both the paramesonephric (Müllerian) and mesonephric (Wolffian) ducts. The Wolffian ducts induce the normal development of the Müllerian ducts in addition to giving origin to the kidneys. Abnormal development of the Wolffian ducts leads to the unilateral renal agenesis and imperforated hemivagina associated with OHVIRA syndrome [2].

This syndrome has been widely recognized due to increasing familiarity with the disorder and improvement in diagnosis [3]. Postpubertal patients are dominant, demonstrating various symptomatic presentations such as cyclic abdominal pain and vaginal discharge, and preserving fertility is a major issue in these patients [4]. Accordingly, previous studies usually focused on postpubertal age group and their proper management. Symptoms occur less frequently in prepubertal patients with this syndrome. Particularly, distended hemivagina presenting as a protruding vaginal mass occurs rarely in neonatal patients and regresses after cessation of exposure to maternal estrogen after birth [5,6]. Due to its rare symptomatic presentation, little is known about OHVIRA in prepubertal group.

Because prenatal ultrasonography is now routinely performed, a large portion of OHVIRA patients are first encountered by urologists evaluating a dysplastic, atrophic, or absent kidney after detection during ultrasonography. Dysplastic or atrophic kidney could be accompanied by an ectopic ureter that connects to the ipsilateral obstructed hemivagina, which can create diagnostic and therapeutic challenges if clinicians are not aware of this possibility [1].

The purpose of this study was to assess the clinical course of prepubertal OHVIRA and elucidate the problems urologists may encounter. By conducting a thorough review of previous studies and our institute’s considerable number of cases, we developed the commonsensical management policy that we propose here. We hope these results will raise awareness of this syndrome in the pediatric urology community.

## Materials and Methods

We carried out a retrospective cohort analysis of patients diagnosed with OHVIRA syndrome at our institution. The study was performed in accordance with applicable laws and regulations, good clinical practices, and the ethical principles described in the Declaration of Helsinki. The study was approved by the Institutional Review Board of Severance Hospital (Approval No. 4-2016-0022). Informed consent was waived due to the retrospective analysis of the study.

### Patient cohort

Between July 2004 and June 2015, 45 prepubertal patients were diagnosed with OHVIRA syndrome before one year of age and followed up for at least six months at our institute. We reviewed their medical records from the time of diagnosis to the most recent follow-up examination. The diagnosis of OHVIRA syndrome was dependent on two features demonstrated by ultrasonography, computed tomography, or magnetic resonance imaging (MRI): 1) Obstructed hemivagina was defined as a distended hemivagina with or without a double uterus. 2) Ipsilateral renal anomalies included renal agenesis and dysplastic or atrophic kidney. Patients with associated cloacal and/or anorectal anomalies and those without sequential imaging data available were excluded from our analysis, resulting in the inclusion of 43 patients.

### Classification of involution type

**Fig 1:**
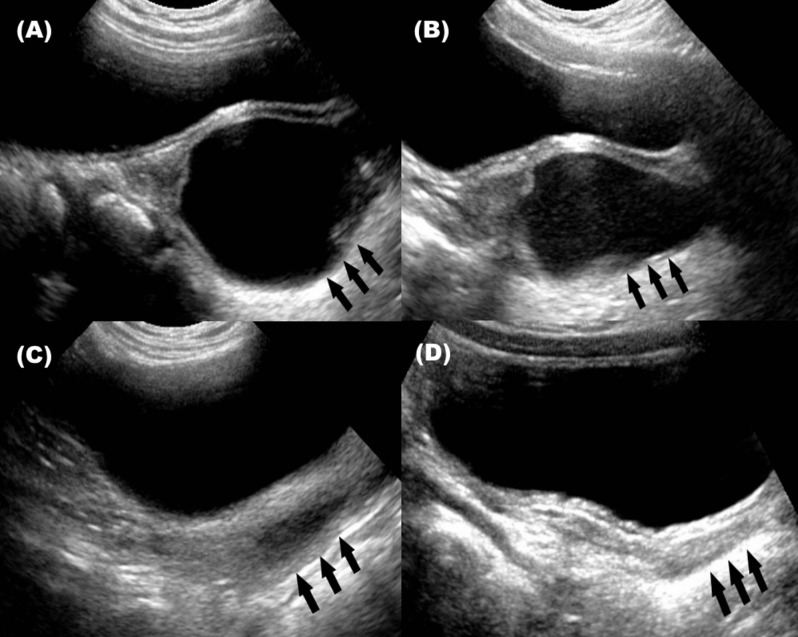
Serial ultrasonography of six-year-old girl who demonstrated complete resolution of hemivaginal distention. (A) Ultrasonography of initial hemivaginal distention performed at 10 months (B),(C) Ultrasonography performed at 16 months and 24 months showing partial resolution of hemivaginal distention (C) Ultrasonography performed at thirty months showing complete resolution. *Arrow head: obstructed hemivagina

The degree of distention in the obstructed hemivagina was assessed sequentially for every patient. The natural course was divided into three types: 1) complete resolution of distention; no residual fluid collection in vagina 2) partial resolution of distention; decrement of fluid collection in vagina, and 3) maintenance or aggravation of distention (Fig 1).

### Accompanying urologic anomalies and problems

Accompanying urologic anomalies were assessed for both the ipsilateral and contralateral sides. During the follow-up period, all the problems that required intervention were assessed for the ipsilateral obstructed hemivagina and the upper urinary tract anomalies. Regarding upper urinary tract anomalies, the problems that were either related to accompanying urologic anomalies or arising from decreased renal function were recorded. Serum creatinine, blood pressure, and urinalysis were evaluated periodically. Creatinine elevation was defined based on the age-adjusted serum creatinine level proposed by Ceriotti et al [7]. Creatinine elevation was diagnosed when the creatinine level was above the 97.5^th^ percentile for age. Hypertension was diagnosed when high blood pressure equal to or greater than the 95^th^ percentile was observed at more than three visits [8]. Normal blood pressure level by age was defined by the Korea Centers for Disease Control and Prevention and the Korean Pediatric Society [8].

### Natural course

Analysis of patients was conducted for two time periods: birth to six months of age, and above six months of age. Six month was selected considering the maximum duration of maternal estrogen effect, causing vaginal bleeding. In each period, the natural courses of Müllerian and Wolffian duct development were analyzed. For patients who required surgical intervention, the age at operation, main reason for the operation, and surgical outcome were described.

## Results

### Patient cohort

The median age at diagnosis was 1.3 months (interquartile range [IQR]: 0.1–3.6) and the median follow-up period was 25.5 months (IQR: 15.3–67.3) (Table 1). Among 43 patients, 10 patients (23.3%) were diagnosed during prenatal imaging process, while 27 patients (62.8%) were diagnosed after birth, at follow-up imaging for ipsilateral renal agenesis or multicystic dysplastic kidney. Four patients (9.3%) were diagnosed via physical exam after birth, and two patients (4.7%) were diagnosed by their symptoms, such as persistent urinary incontinence and urinary tract infection. Occurrence was approximately equal on the left and right sides. Uterine anomalies included uterine didelphys in the greatest percentage of patients (86.7%, 38/43) followed by bicornuate uterus (6.7%, 2/43). Dysplastic kidney was a more commonly observed kidney anomaly than renal agenesis (28 and 15 cases, respectively). For the 29 dysplastic kidney patients, ectopic MCDK was more commonly observed than orthotopic MCDK, and some cases were initially diagnosed as renal agenesis by prenatal sonography. Obstructed hemivagina distention was resolved during the follow-up period in approximately the same number of cases as it was maintained; it completely resolved in 12 patients, partially resolved in 10, and was maintained or aggravated in 21. Renal function deterioration was observed in 5% (2/43), and surgery was required in 13% (6/43) with a median age at operation of 31 months (Table 1).

**Table 1:** Baseline patient characteristics.

Characteristics	
Number of patients, N (%)	43 (100)
Age at diagnosis [months], median (IQR)	1.3 (0.1–3.6)
Follow-up period [months], median (IQR)	25.5 (15.3–67.3)
Laterality [R:L], N (%)	22:21 (51.2:48.8)
Uterine anomaly, N (%)	
Single uterus	3 (7.0)
Didelphys	38 (88.4)
Bicornuate	2 (4.7)
Kidney anomaly, N (%)	
Multicystic dysplastic kidney	28 (65.1)
Renal agenesis	15 (34.9)
Clinical course, N (%)	
Complete resolution of vaginal distention	12 (27.9)
Partial resolution of vaginal distention	10 (23.3)
Maintenance or aggravation of vaginal distention	21 (48.8)
Renal function deterioration^a^, N (%)	2 (4.7)
Surgery, N (%)	6 (14.0)
Age at operation [months], median (IQR)	31.2 (14.0–50.9)

^a^ Elevated age-adjusted creatinine [7]

### Associated urologic anomalies

When ipsilateral urologic anomalies were considered, ectopic ureter insertion to the vagina was most frequently observed (62.8%, 27/43), followed by ureterocele and vesicoureteral reflux (VUR). On the contralateral side, VUR was the most commonly observed anomaly (7.0%, 3/43), followed by megaureter and dysplastic kidney.

### Natural course and Operation

#### 1. Birth to six months (N = 43)

**Fig 2:**
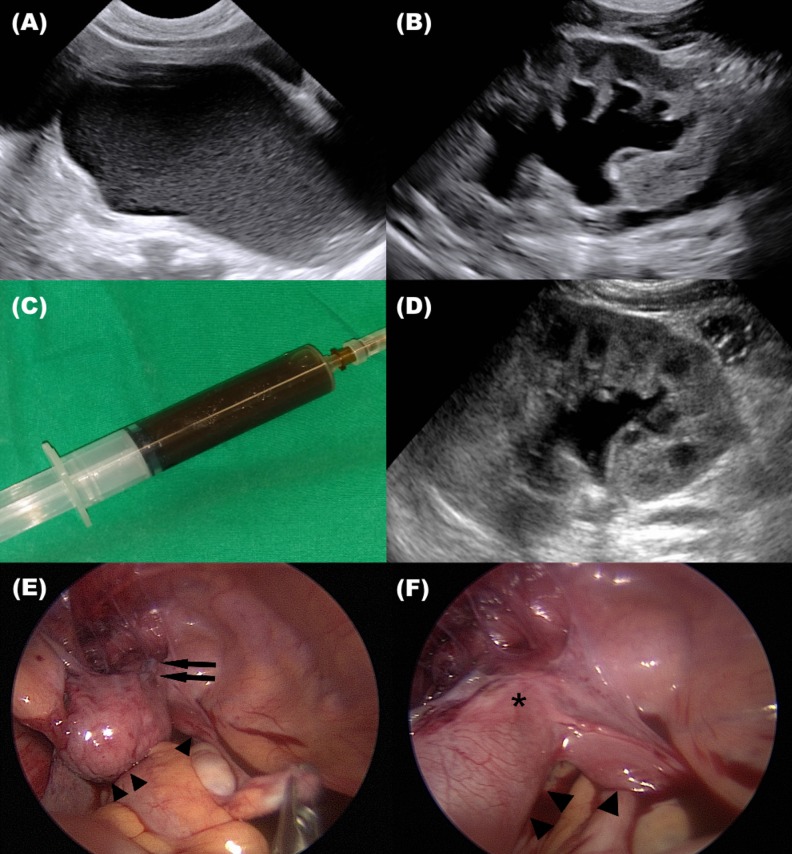
Representative images of two cases that underwent operation. (A) Ultrasonography of a two-month-old girl with fluid-filled vaginal distention. (B) Contralateral hydronephroureterosis due to pressure from the vaginal mass. (C) Aspirated hemorrhagic vaginal fluid. (D) Resolution of hydronephroureterosis after vaginal fluid aspiration. (E, F) Laparoscopic features of a four-year-old girl with obstructed hemivagina and ipsilateral ureter insertion to hemivagina. * Arrow head: bilateral uterus, Arrow: ipsilateral ureteral stump, Asterisk: ipsilateral obstructed hemivagina.

One out of the 43 patients (2.3%) required an operation due to a very large vaginal mass compressing the contralateral urinary tract, leading to grade 2 hydroureteronephrosis. Vaginal aspiration was performed at 2 months (Table 2); the aspirated fluid was hemorrhagic fluid originating from neonatal vaginal bleeding. Ultrasonography was performed one week postoperatively and the contralateral hydronephrosis had improved as the size of the vaginal mass was reduced (Fig 2). Six patients (14.0%) showed complete resolution of vaginal distention within their first six months. One patient demonstrated a reduced glomerulation filtration rate of 22.7 mL/min/1.73m^2^ due to their accompanying contralateral dysplastic kidney.

**Table 2:** Characteristics of six patients who required operation.

No.	MCDK/Agenesis	Laterality	Age at diagnosis (months)	Age at operation (months)	Follow-up period (months)	Type of surgery	Reason for surgery	Result
1	Agenesis	Right	At birth	2.1	3.4	Vaginal septum resection, and hemorrhagic fluid aspiration	Secondary contralateral hydroureteronephrosis due to huge obstructed hemivagina	Improved contralateral hydroureteronephrosis, Renal function preservation
2	Ectopic MCDK	Right	2.1	12.5	52.4	Nephrectomy, Vaginal septum resection and clear fluid aspiration	Obstructed hemivagina with very large outward protrusion	No vaginal distention, No urinary incontinence
3	MCDK	Left	2.7	18.4	134.7	Nephrectomy	Recurrent urinary tract infection	No vaginal distention, No urinary incontinence, No urinary tract infection
4	MCDK	Left	At birth	23.6	87.2	Vaginal septum resection and clear fluid aspiration	Obstructed hemivagina with very large outward protrusion	Urinary incontinence
				44.0	87.2	Nephrectomy	Urinary incontinence	No vaginal distention, No urinary incontinence
5	Ectopic MCDK	Right	3.5	44.1	60.6	Nephrectomy	Urinary incontinence	No vaginal distention, No urinary incontinence
6	Ectopic MCDK	Left	1.7	53.2	81.2	Nephrectomy, Vaginal clear fluid aspiration	Abdominal pain	No abdominal pain, No vaginal distention

MCDK: multicystic dysplastic kidney

#### 2. After six months (N = 36)

Excluding the seven patients who underwent surgery or showed complete resolution of vaginal distention before six months of age, vaginal distention continued past six months of age in 36 patients. Among those, five patients (13.9%) presented complicated symptomatic presentations such as protruding vaginal mass, recurrent urinary tract infection, urinary incontinence, and abdominal pain, requiring surgery (Table 2). Five patients underwent ipsilateral nephrectomy, two of which required concurrent vaginal septum resection. The fluid aspirated from these patients was clear, highly suggestive of urine. One patient initially underwent solely vaginal septum resection with clear fluid aspiration, but later required ipsilateral nephrectomy due to urinary incontinence. All the patients who underwent aspiration demonstrated ectopic ureter insertion into the vagina (Fig 2). Postoperatively, no vaginal distention or urinary tract infections were observed. Six of the 36 patients (16.7%) showed complete resolution and seven patients (19.4%) showed partial resolution of vaginal distention. One patient demonstrated persistently elevated creatinine adjusted for age, suggesting continued renal function deterioration.

## Discussion

Most OHVIRA patients are diagnosed months after menarche as a result of symptomatic presentation, commonly including hematocolpos, pelvic pain, vaginal or pelvic mass, and sometimes including abnormal vaginal discharge, abdominal pain, vomiting, fever, or acute urinary retention (AUR) [9,10]. These symptomatic cases usually require surgical intervention such as full excision and marsupialization of the vaginal septum for the purpose of preventing complications and preserving fertility [9,11]. However, as prenatal ultrasonographic screening has become routine, the age at diagnosis has decreased for congenital malformations. Thus far, only a few reports regarding prepubertal OHVIRA patients have been published (Table 3). Only 20 cases have been reported, with ten cases showing ipsilateral renal agenesis and the other ten ipsilateral MCDK [5,6,10,12–17]. Among these 20 cases, abdominal pain occurred in three patients (15%), abdominal or vaginal mass occurred in 12 (60%), and pyocolpos and AUR each occurred in two cases (10%).

**Table 3:** Characteristics of patients requiring operation in the previous studies.

No.	Author	MCDK/Agenesis	Laterality	Age at operation (months)	Type of surgery	Reason for surgery	Result
1	Pansini et al. [10]	Ectopic MCDK	Right	5.0	Nephrectomy, Hemorrhagic fluid aspiration	AUR caused by protruding mass	No vaginal distention
2	Capito et al. [14]	MCDK	Left	9.0–10.0	Nephrectomy	Vulvar mass	N/A
3		MCDK	Right	0.0–1.0	Nephrectomy	Vulvar mass	N/A
4		MCDK	Right	0.0–1.0	Nephrectomy	Vulvar mass	N/A
5		MCDK	Left	0.0–1.0	Nephrectomy	Vulvar mass	N/A
6		Agenesis	Right	0.0–1.0	Nephrectomy	Vulvar mass	N/A
7		Agenesis	Left	6.0–7.0	Nephrectomy	Vulvar mass	N/A
8		Agenesis	Right	14.0–15.0	Nephrectomy	AUR, Abdominal mass, Pyocolpos	N/A
9		Agenesis	Right	84.0	Nephrectomy	Abdominal pain, Abdominal mass	N/A
10	Roth et al. [15]	Agenesis	Left	36.0	Vaginal septum resection	Painless abdominal mass	No vaginal distention
11	Vivier et al. [16]	Agenesis	Left	60.0	Vaginal septum resection and clear fluid aspiration	None	No vaginal distention
12	Sanghvi et al. [6]	Agenesis	Right	48.0	Abdominal exploration through Pfannenstiel incision and clear fluid aspiration	Abdominal pain	Symptom-free
13	Kiechl-Kohlendorfer et al. [12]	MCDK	Right	28.0	Vaginal septum resection	Pyocolpos	N/A
14	Wu et al. [17]	Agenesis	Right	0.2	Transhymenal incision and fluid drainage	Protruding vaginal mass	No vaginal distention
15	Han et al. [5]	Agenesis	Left	N/A	N/A	N/A	N/A
16	Angotti et al. [13]	Agenesis	Right	36.0	Vaginal septum resection and mucous drainage	Abdominal pain, Abdominal mass	Symptom free

MCDK: multicystic dysplastic kidney, AUR: acute urinary retention, N/A: not available

Among the 20 previously published cases, eight patients (40%) also experienced ectopic ureter insertion into the vagina, causing hydrocolpos and related problems. In our study, more than half of the prepubertal patients were suspected to have accompanying ectopic ureter insertion into the vagina, and the majority of these demonstrated maintenance or aggravation of vaginal distension. In 2010, Wang et al. reviewed the previous reports of pubertal girls and concluded that ectopic ureter associated with this syndrome is very rare, because only two cases had been reported at that time compared to 20 patients without ectopic ureter insertion [18]. We believe this discordance was due to different inclusion criteria being used in the previous studies. Specifically, we included patients with ipsilateral dysplastic kidney, which our previous study showed is misdiagnosed as renal agenesis by ultrasonography in about half of patients [19]. In the current study, we included patients with ipsilateral multicystic dysplastic kidney as well as renal agenesis, while only about 50% of previous studies made such inclusion. According to Carrico *et al*., defining the presence of ectopic ureter radiologically in dysplastic kidney is difficult before toilet training [20], since the ureter usually is non-dilated and hidden by the surrounding tissue. For this reason, we admit that the incidence rate could have been different.

**Fig 3:**
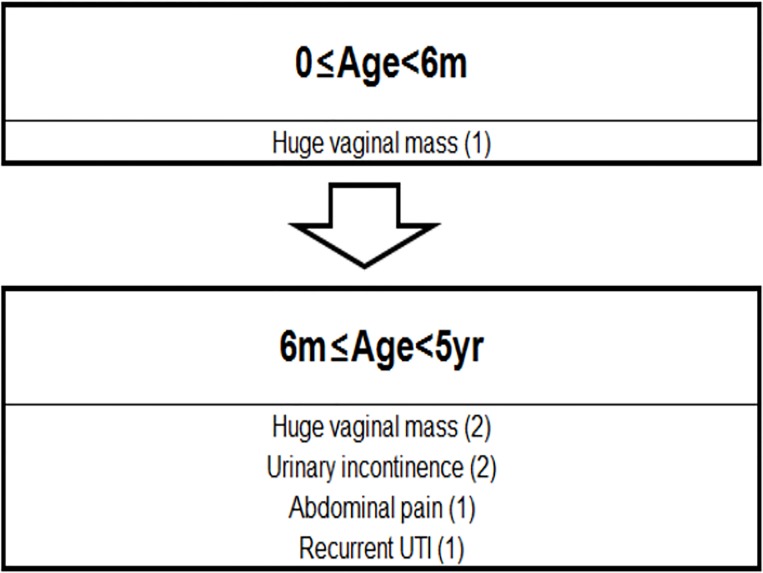
A diagram summarizing problems that require surgery in our study, according to patient age. *UTI: urinary tract infection

We arbitrarily divided the analysis of patients into two time periods: from birth to six months of age, and after six months of age (Fig 3). This cutoff was selected because maternal estrogen may cause accumulation of vaginal secretion or vaginal bleeding during the first six months of life. Neonatal vaginal bleeding is known to last for about ten days after birth [21], and Pansini et al. demonstrated that estrogen is present in the serum of newborns for at least six months after delivery and could stimulate secretion in target epithelia [10]. Indeed, the patient requiring vaginal aspiration before one year of age showed hemorrhagic fluid in the vagina, while clear fluid suggestive of urine was aspirated from the older patients. All patients over one year old who required surgery exhibited ectopic ureter insertion into the vagina. The rate of resolution of vaginal distention was also lower in the older patients. This suggests that while vaginal bleeding may resolve earlier, if urine accumulates in the obstructed hemivagina due to ectopic ureter insertion, resolution may be relatively slow, causing symptomatic presentation or problems. In the younger age group, the dominant reason for surgical intervention was a vaginal mass protruding outward. However, as the patients grow older, problems occurred because of long-term accumulation of vaginal fluid, such as hydropyocolpos, abdominal pain, and recurrent urinary tract infection. Based on our age classification, we grouped the patients described in previous studies and analyzed the results. Similar to our study, in the group less than one year of age, severe vaginal mass was the main reason for operation, while in the older group, abdominal pain was the most common reason. Nine patients (60%) required ipsilateral nephrectomy, while a vaginal approach was sufficient for six patients (40%).

**Fig 4:**
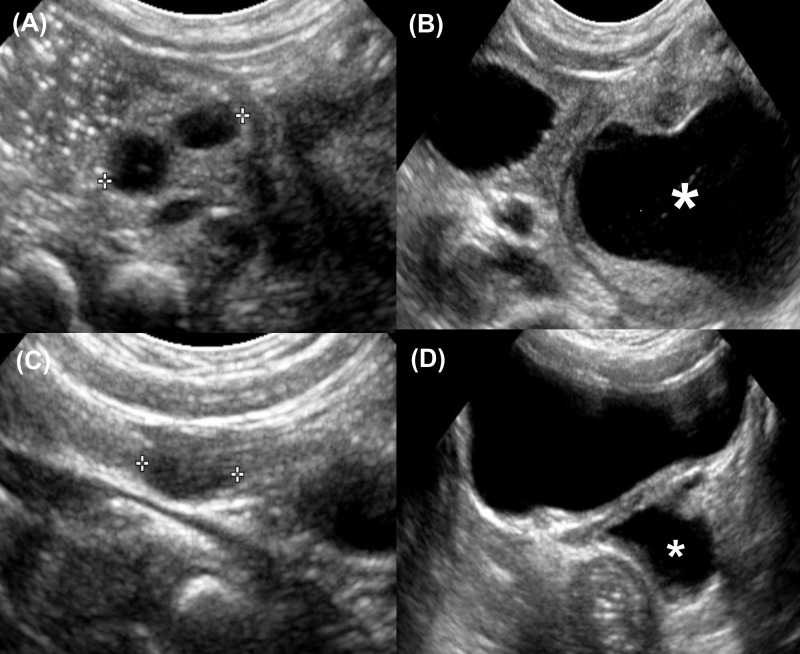
Natural course of OHVIRA syndrome with ectopic ureter insertion into obstructed hemivagina. (A),(B) Two days ultrasonography demonstrating 1.4cm sized ipsilateral ectopic multicystic dysplastic kidney(MCDK) and distended hemivagina. (C),(D) 14months ultrasonography demonstrating decreased size of ectopic MCDK (0.8cm) with decreased sized of distended hemivagina. *Asterisk: obstructed hemivagina

For the patients who did not undergo surgery, the median follow-up period was 24.6 months (IQR: 14.0–56.5). About 30% experienced complete resolution of vaginal distention, with a median time to resolution of about four months. Another 25% demonstrated reduced vaginal distention (Fig 4). The other 45% showed persistent vaginal distention, but without symptoms or surgical indications. The longest period of observed persistent distention was 68.6 months. We can infer that roughly half of the patients still may require operation in the future due to problems resulting from long-term fluid accumulation, such as abdominal pain or pyocolpos. Therefore, follow-up imaging is still necessary throughout the prepubertal period in case of persistence of vaginal distention.

For patients who did undergo surgery, the median age at operation was 31.2 months (IQR: 14.0–50.9). Among the patients from previous studies, the median age at operation was 9.5 months (IQR: 0.5–36.0). Including our patients, a total of 22 prepubertal OHVIRA patients requiring surgical intervention have been reported so far. All patients except one, who required operation due to abdominal pain at 84 months, demonstrated problems before the age of five. Most of the cases in our series with suspected urine collection in the obstructed hemivagina due to ectopic ureter insertion were not definitely defined in the preoperative imaging studies. However, in all cases in our series, nephrectomy alone resolved the main symptoms such as recurrent urinary tract infection, urinary incontinence, and abdominal pain and also resolved the vagina distention in the following imaging studies as shown in Table 2. We have not performed vaginal septum resection routinely, because the resected septum may not be maintained as patients undergo puberty for there is no long term outcome study reported yet. At pubertal age, septum resection in that time may be the right choice with higher success rate and lower recurrence rate in our perspective. According to Joseph *et al*., there is a 15–20% chance of recurrence after vaginal septum resection, possibly due to incomplete resection resulting from a narrow operative field as well as the risks of re-epithelialization, fibrosis, and adhesion formation [22].

The management of a single kidney should not be overlooked. In this study, about 15% of patients had accompanying contralateral urologic anomalies, with VUR being the most common, raising concern for damage to the solitary kidney. Among three contralateral VUR cases, two patients experienced febrile urinary tract infections. The patient experiencing contralateral dysplastic kidney demonstrated chronic kidney disease at an early age.

For the same period time, 1.9% (43/2227) of female patients with multicystic dysplastic kidney or renal agenesis diagnosed as at our institution was found out to have OHVIRA syndrome. Due to this relatively low incidence rate, it is not worth spending the increased cost to screen everyone for OHVIRA syndrome. Perhaps a basic renal and bladder ultrasound can be used to find signs suggestive of dilation behind the bladder, followed by a formal pelvic ultrasound to evaluate the results. Still, however, clinicians should always consider the possibility of this diagnosis and be awake of the patients’ symptoms related with both Müllerian and Wolffian ducts anomalies.

Based on our results and our analysis of previous findings, once patient is diagnosed as OHVIRA, prepubertal patients require regular follow-up before five years of age. First, the vaginal mass should be physically examined and any newly developed symptoms must be recorded. The symptoms may vary widely, as demonstrated in previous reports as well as the present study. Second, regular ultrasonography is needed to monitor vaginal distention, with consideration of the patient’s age, the degree of vaginal distention, and the status of contralateral solitary kidney. While similar management techniques are used among prepubertal patients (over the age of five years), the process is quite different for postpubertal patients. For instance, vaginal septotomy and vaginoplasty are used as standard care in postpubertal patients, rather than ipsilateral nephrectomy, because hemorrhagic fluid collection is the problem that usually arises from menstruation. Furthermore, preserving fertility is another big issue during the menstrual period [3,14].

For patients showing early vaginal distention before one year of age, there is a possibility of hemorrhagic fluid collection that can lead to dynamic changes of vagina distention. Early-onset problems requiring intervention may occur; or, on the contrary, rapid resolution may happen as well. However, when considering intervention through vaginal approach due to hemorrhagic fluid collection, one should still be cautioned about the possibilities of either ectopic ureter insertion or a required ipsilateral nephrectomy in the future. In patients showing relatively latent and persistent vaginal distention, there is a high likelihood of urine collection, which may lead to problems gradually resulting from time-dependent accumulation of urine. In addition, renal function should also be evaluated over a similar period, especially for patients with urologic anomalies that can place a single kidney at risk.

Our study has some limitations. First, this was a retrospective study, which presents potentials for selection bias. The study also included a small number of subjects, although it is presently the largest single-institution study. The data related to complications are still too limited for either accurate analysis of the development of high blood pressure or assessment of renal function. Moreover, our initial interest in this study focused on ipsilateral renal anomalies, which can lead to insufficient information related to other accompanying Mullerian anomalies, rather than on obstructed hemivagina. Further studies are required to consider the yield of routine screening for Mullerian anomalies, in order to assess the accompanying ipsilateral renal anomalies.

## Conclusions

OHVIRA syndrome is known as a postpubertal disease; however, about 13% of prepubertal patients examined required surgery. When accompanied by ectopic ureter insertion into the vagina, continuous urine production may necessitate further treatment. The development of complications should be monitored during the follow-up period, especially before five years of age.

## Supporting Information

S1 File Raw data of study cohort. (XLSX)
